# Supplementary figures and images for: Gender differences in the distribution of children’s physical activity: evidence from nine countries
Source: Int J Behav Nutr Phys Act. 2023 Sep 4;20:103. doi: 10.1186/s12966-023-01496-0 (PMC10478357; doi:10.1186/s12966-023-01496-0)

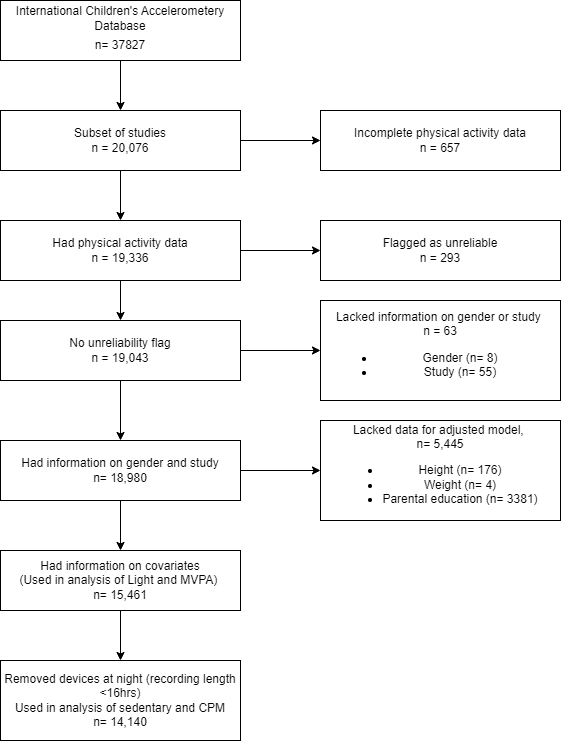

Supplement: Supplementary file 1 — Additional file 1. [file 12966_2023_1496_MOESM1_ESM.zip › 12966_2023_1496_MOESM1_ESM/12966_2023_1496_FigS1_Print.png]

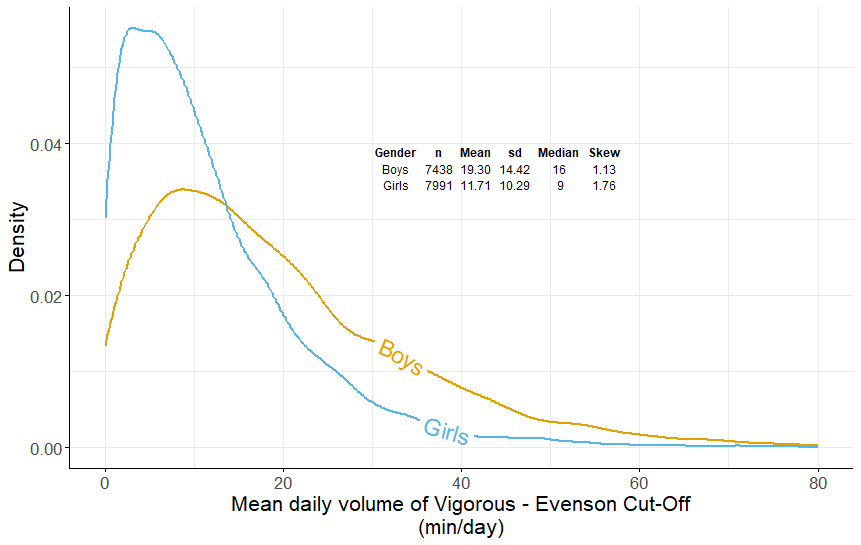

Supplement: Supplementary file 1 — Additional file 1. [file 12966_2023_1496_MOESM1_ESM.zip › 12966_2023_1496_MOESM1_ESM/12966_2023_1496_FigS10_Print.png]

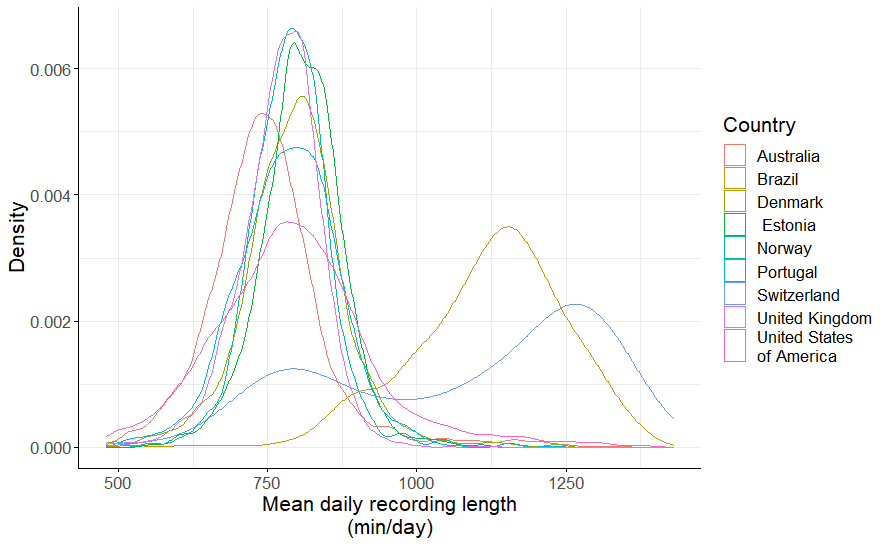

Supplement: Supplementary file 1 — Additional file 1. [file 12966_2023_1496_MOESM1_ESM.zip › 12966_2023_1496_MOESM1_ESM/12966_2023_1496_FigS2_Print.png]

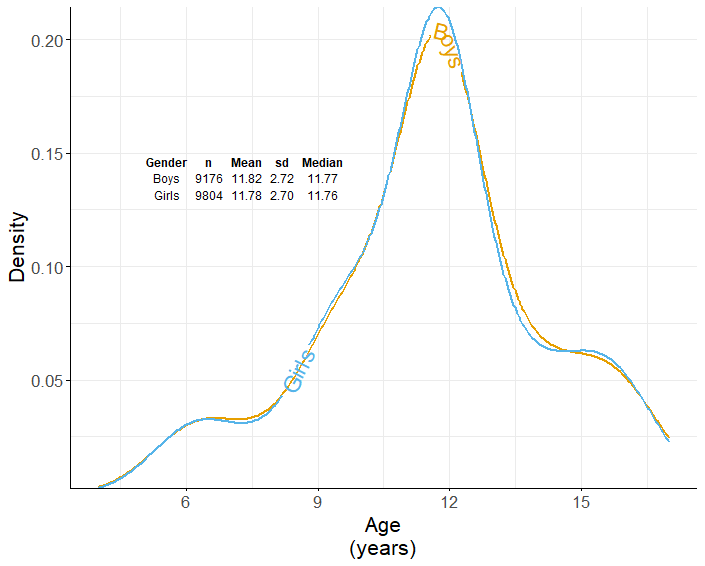

Supplement: Supplementary file 1 — Additional file 1. [file 12966_2023_1496_MOESM1_ESM.zip › 12966_2023_1496_MOESM1_ESM/12966_2023_1496_FigS3_Print.png]

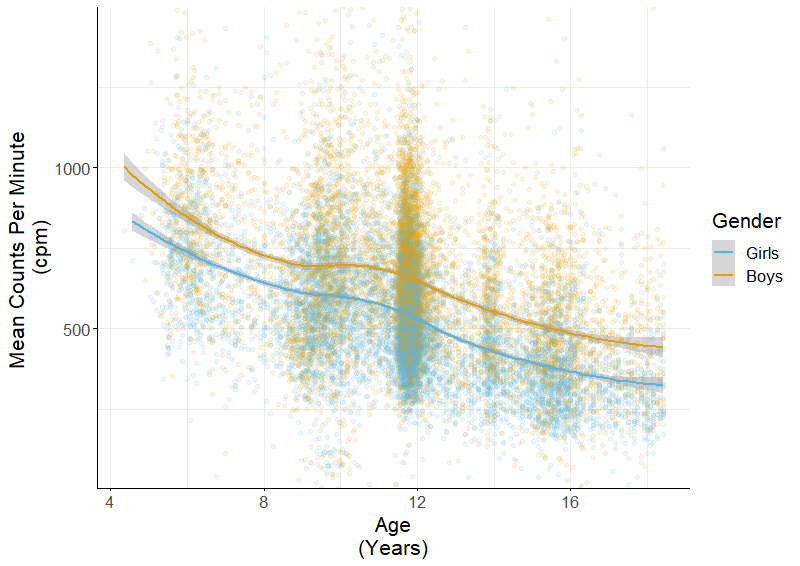

Supplement: Supplementary file 1 — Additional file 1. [file 12966_2023_1496_MOESM1_ESM.zip › 12966_2023_1496_MOESM1_ESM/12966_2023_1496_FigS4_Print.png]

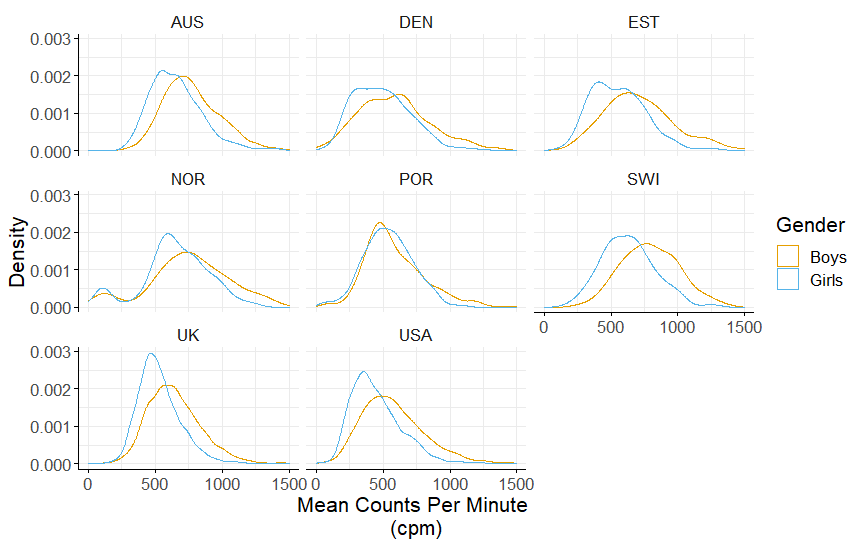

Supplement: Supplementary file 1 — Additional file 1. [file 12966_2023_1496_MOESM1_ESM.zip › 12966_2023_1496_MOESM1_ESM/12966_2023_1496_FigS5_Print.png]

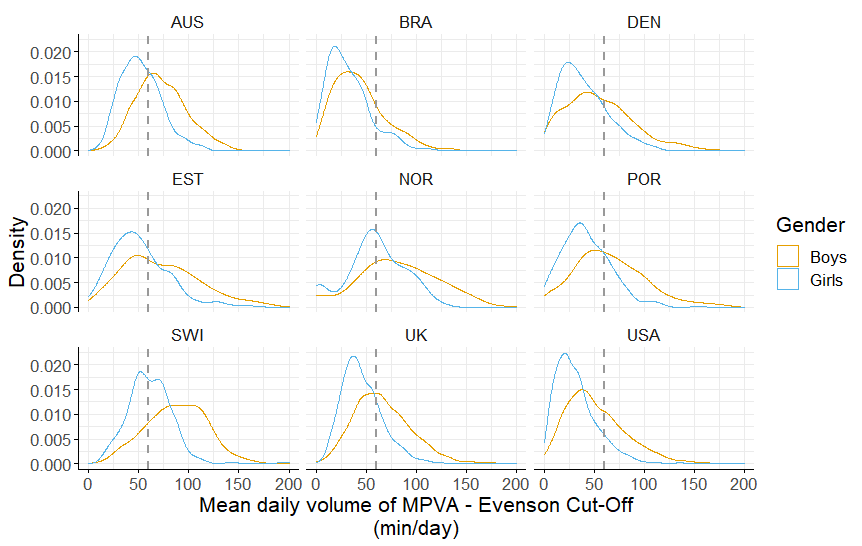

Supplement: Supplementary file 1 — Additional file 1. [file 12966_2023_1496_MOESM1_ESM.zip › 12966_2023_1496_MOESM1_ESM/12966_2023_1496_FigS6_Print.png]

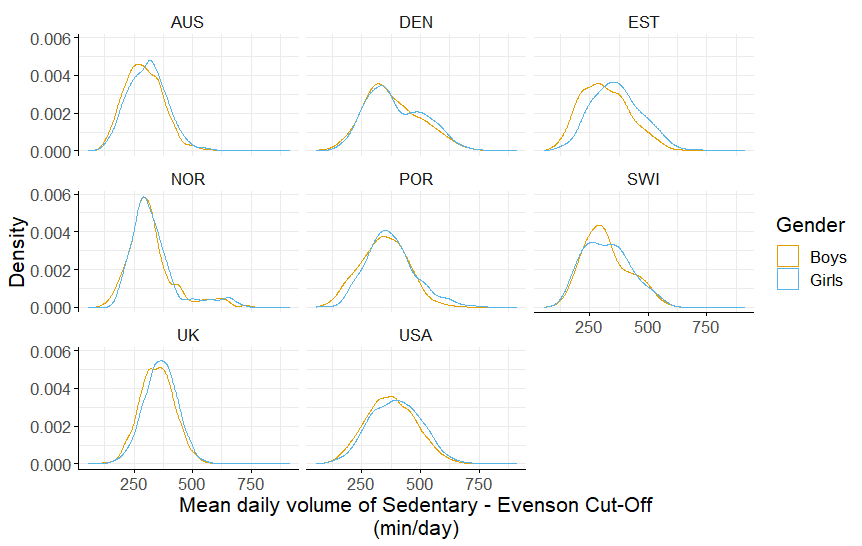

Supplement: Supplementary file 1 — Additional file 1. [file 12966_2023_1496_MOESM1_ESM.zip › 12966_2023_1496_MOESM1_ESM/12966_2023_1496_FigS7_Print.png]

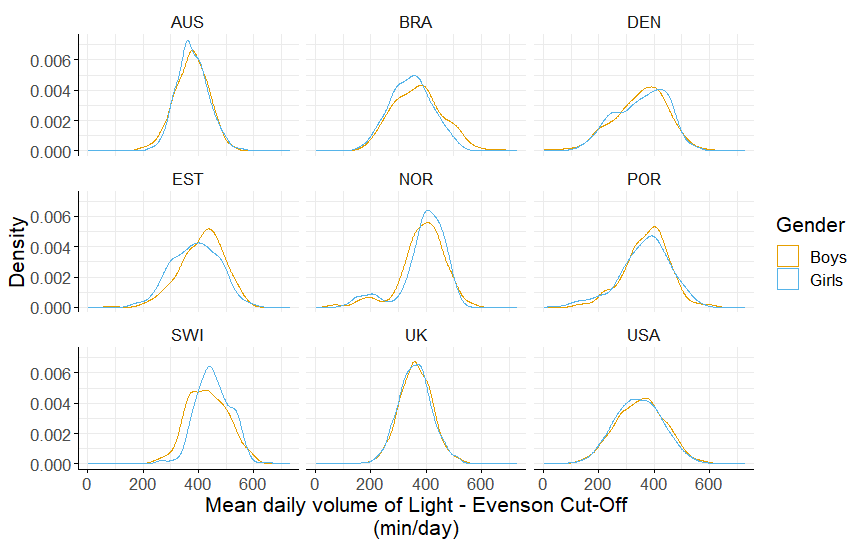

Supplement: Supplementary file 1 — Additional file 1. [file 12966_2023_1496_MOESM1_ESM.zip › 12966_2023_1496_MOESM1_ESM/12966_2023_1496_FigS8_Print.png]

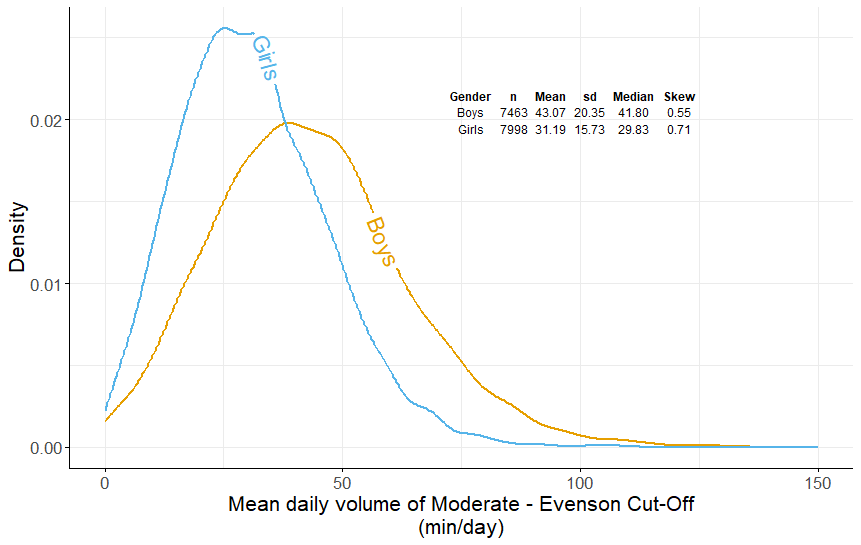

Supplement: Supplementary file 1 — Additional file 1. [file 12966_2023_1496_MOESM1_ESM.zip › 12966_2023_1496_MOESM1_ESM/12966_2023_1496_FigS9_Print.png]
